# A Motor Imagery Signals Classification Method *via* the Difference of EEG Signals Between Left and Right Hemispheric Electrodes

**DOI:** 10.3389/fnins.2022.865594

**Published:** 2022-05-09

**Authors:** Xiangmin Lun, Jianwei Liu, Yifei Zhang, Ziqian Hao, Yimin Hou

**Affiliations:** ^1^School of Automation Engineering, Northeast Electric Power University, Jilin, China; ^2^School of Finance and Business, Jinan Vocational College, Jinan, China

**Keywords:** brain-computer interface (BCI), electroencephalography (EEG), motor imagery (MI), convolutional neural network (CNN), weighted minimum norm estimation (WMNE)

## Abstract

Brain-computer interface (BCI) based on motor imagery (MI) can help patients with limb movement disorders in their normal life. In order to develop an efficient BCI system, it is necessary to decode high-accuracy motion intention by electroencephalogram (EEG) with low signal-to-noise ratio. In this article, a MI classification approach is proposed, combining the difference in EEG signals between the left and right hemispheric electrodes with a dual convolutional neural network (dual-CNN), which effectively improved the decoding performance of BCI. The positive and inverse problems of EEG were solved by the boundary element method (BEM) and weighted minimum norm estimation (WMNE), and then the scalp signals were mapped to the cortex layer. We created nine pairs of new electrodes on the cortex as the region of interest. The time series of the nine electrodes on the left and right hemispheric are respectively used as the input of the dual-CNN model to classify four MI tasks. The results show that this method has good results in both group-level subjects and individual subjects. On the Physionet database, the averaged accuracy on group-level can reach 96.36%, while the accuracies of four MI tasks reach 98.54, 95.02, 93.66, and 96.19%, respectively. As for the individual subject, the highest accuracy is 98.88%, and its four MI accuracies are 99.62, 99.68, 98.47, and 97.73%, respectively.

## 1. Introduction

The electroencephalogram (EEG) signal is the electrical activity of neurons in the brain recorded by EEG sensors. It has high temporal resolution and low spatial resolution (Nakamura et al., 2005). Currently, motor imagery EEG (MI-EEG) has received widespread attention because it can decode motion intention (Pfurtscheller et al., 2006). The brain-computer interface (BCI) can detect the intention of the MI-EEG signal and convert it into an executable output by the machine (Millan and Del, 2002). In other words, it can communicate with external devices by decoding MI tasks, so as to achieve two-way feedback between the user and the BCI system. The external device receives signals from the brain to control the device, and the device feeds back the control results to the brain for judgment (Jin et al., 2020). MI-BCI can help some disabled patients independently control external devices such as wheelchairs (Wang and Bezerianos, 2017) and artificial limbs (Condori et al., 2016; Cho et al., 2019). Bhattacharyya et al. (2021) designed a real-time BCI neurofeedback system to reflect the expected tasks of hand movement and imagery.

Effective feature extraction can achieve high-precision decoding on MI-BCI. Entropy and sensor-imotor rhythm (SMR) are currently popular features in MI-BCI. In SMR-based BCI, He et al. (2015) reviewed the principles and approaches of developing an SMR EEG based BCI and found that the SMR based noninvasive BCI has the potential to provide communication and control capabilities. Yuan and He (2014) described the characteristic features of SMR from the human brain and discussed their underlying neural sources, also reviewed the functional components of SMR-based BCI, together with its current clinical applications. Serafeim et al. (2018) trained two severely impaired participants with chronic spinal cord injury (SCI) following mutual learning approach in a virtual BCI race game, it substantiates the effectiveness of this type of training. In entropy-based BCI, Stefano et al. (2019) proposed a novel approach based on the entropy of the EEG signals to provide a continuous identification of motion intention. The result shows that the proposed system can be used to predict motion in real-time at a frame rate of 8 Hz with 80 ± 5% of accuracy. Lei et al. (2012) extracted the sample entropy of the EEG and used support vector machines for pattern classification, it is found that sample entropy can effectively distinguish the characteristics of the brain in different states. Hsu (2015) extracted wavelet fuzzy approximate entropy and used SVM for classification, the results indicate that the proposed system including wavelet-based fuzzy approximate entropy (wfApEn) obtains better performance in average classification.

Recently, there have been many studies on the cortex. Hou et al. (2019) created ten regions of interest in the cortex and performed a time-frequency analysis on them. Edelman B. et al. (2015) explored the cortex dynamics during movements of an unaffected body part in tetraplegic subjects with chronic spinal cord injury. Kim and Kim (2018) analyzed the motor cortex of primates and provided an effective method to decode invasive BCI.

A convolutional neural network (CNN) is a practical tool in many fields, such as image classification (Krizhevsky et al., 2017), sentence classification (Kim, 2014), and EEG decoding (Schirrmeister et al., 2017). It reduces the data preprocessing steps and manual feature processing steps. Also, deep learning has made outstanding contributions to the improvement of MI-BCI (Li et al., 2018; Zhang et al., 2018; Cho et al., 2019; Robinson et al., 2019). Nakagome et al. (2020) used neural networks and machine learning algorithms to decode EEG. The results indicate that neural networks are of great significance in the decoding of EEG signals. Tortora et al. (2020) used a trained long short term memory deep neural network to decode EEG gait, and the proposed decoding method obtains more than 90% robust reconstruction. Al-Saegh et al. (2021) gathered 40 related articles on deep neural network architecture and MI-EEG tasks, and the results show that deep neural networks play a positive role in MI-EEG classification.

Brain-computer interface is a technology that reads EEG signals, records and decodes brain activities, manipulates the activities of specific brain regions, and affects its functions. Based on this, accurate decoding of EEG signals is very important for BCI systems. Since EEG signal is dynamic time series data with a low signal-to-noise ratio, the decoding accuracy of EEG signals has always been a challenge. Although many scholars have made remarkable achievements in this field, there is still a gap between the BCI system and practical application standards, and there is still much room for improvement in the classification method and accuracy of EEG signals.

The contributions of this article are summarized as follows: In this article, we proposed a MI signals classification method *via* the difference of EEG signals between left and right hemispheric electrodes. Based on the Physionet database, the EEG signal on the scalp layer is inversely mapped to the cortex of the brain, and then 9 pairs of new electrode pairs are created, which contain higher SNR information. The time-frequency analysis method is used to extract feature information from the time and frequency series of cortical electrodes. The dual-CNN model proposed in this article has the same settings, including 4 layers of CNN for learning EEG features, 4 layers of max pooling for dimension reduction, a Flatten operation for converting multidimensional data into one-dimensional data, and 1-layer fully connected (FC) layer for classification. This method combines the electrode channel information of the symmetrical regions of interest on the left and right hemispheres of the cortex with the CNN, which realizes the high-precision classification task and provides a new idea for simplifying the design of the BCI system.

The remainder of this article is organized as follows: Section 2 is the Materials and Methods. Section 3 is the Classification Accuracies of the Subjects. Section 4 is a Discussion. Finally, Section 5 is the Conclusion of this article.

## 2. Materials and Methods

### 2.1. The Framework

**Figure 1:**
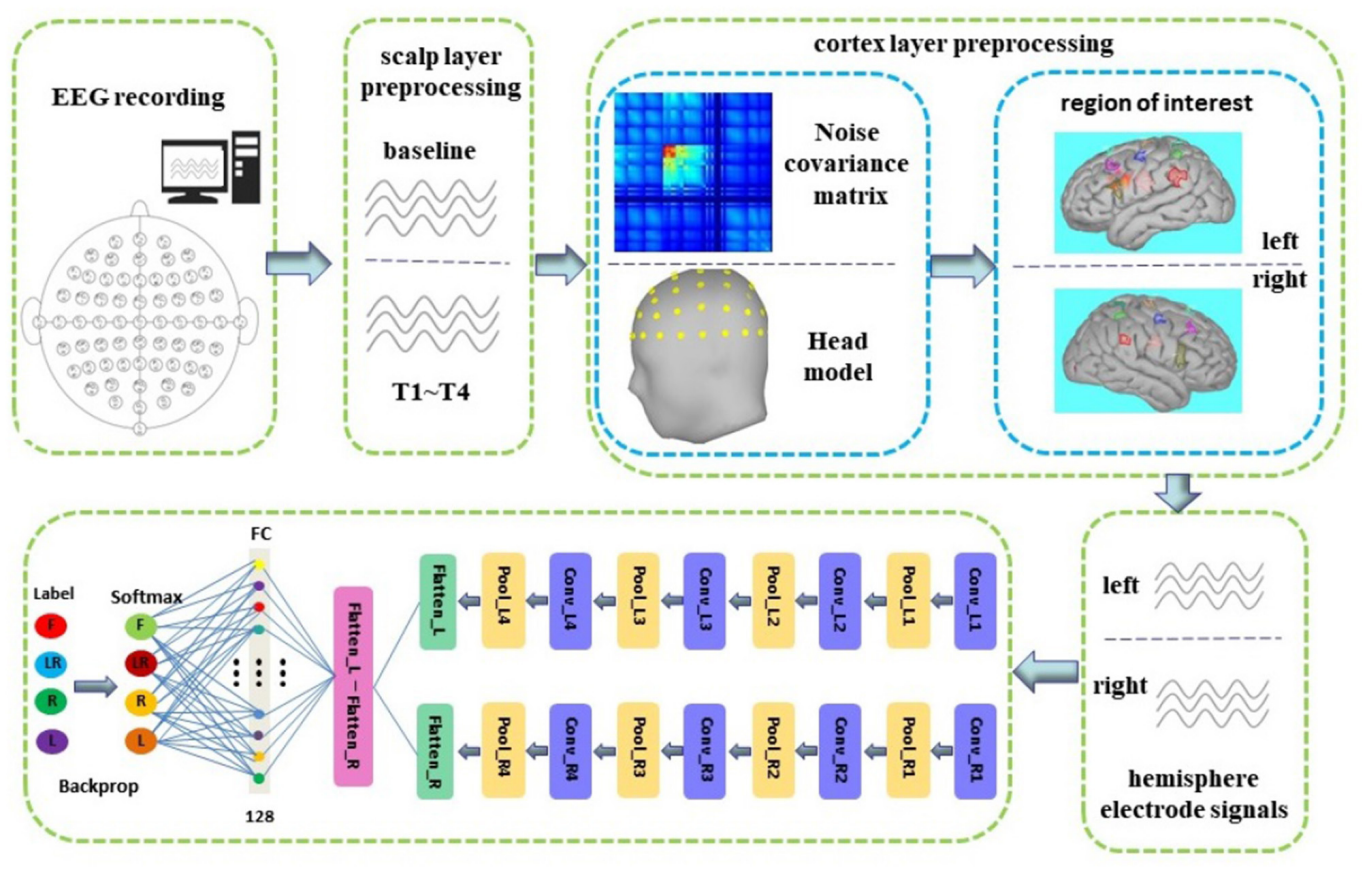
The framework of the proposed approach.

The overall block diagram is shown in Figure 1. In this study, we first preprocessed the EEG on the scalp layer and then preprocessed it on the cortex layer. The noise covariance matrix of each subject was calculated in the cortex preprocessing, and the real head model was constructed with the help of the colin27 template and BEM algorithm. We used distributed current model (DCD) and WMNE algorithm to build a source model and then limited the source to the cortex layer. Then, manually created nine pairs of new electrodes on the left and right hemispheric of the cortex. Finally, the time series carried by the nine pairs of electrodes on the left and right hemispheric were used as the input of the dual-CNN classification model. The preprocessing process and the CNN model structure will be introduced in detail in the following Sections 2.3 and 2.4.2.

### 2.2. Dataset

The dataset we used was created by the developers of BCI2000 at a sampling frequency of 160Hz in a standard environment (Schalk et al., 2004). It is obtained through the corresponding 10-10 system 64-channel EEG (excluding electrodes Nz, F9, F10, FT9, FT10, A1, A2, TP9, TP10, P9, and P10). The dataset records 4 MI tasks (left fist, right fist, both fists, and both feet) of 109 subjects. Each subject consists of 84 trails with 21 trails per class, each trail takes 1–2 min, and the duration of each MI task is slightly more than 4 s. The four tasks are as follows:

1. A target appears on the left side of the screen. The subject imagines opening and closing the left fist until the target disappears. Then the subject relaxes.
2. A target appears on the right side of the screen. The subject imagines opening and closing the right fist until the target disappears. Then the subject relaxes.
3. A target appears on the top of the screen. The subject imagines opening and closing both fists until the target disappears. Then the subject relaxes.
4. A target appears on the bottom of the screen. The subject imagines opening and closing either both feet until the target disappears. Then the subject relaxes. Detailed data description is given at https://archive.physionet.org/pn4/eegmmidb/.

### 2.3. Data Preprocessing

Data preprocessing is divided into two parts: scalp layer preprocessing and cortex layer preprocessing. In the scalp layer preprocessing, we marked 4 MI tasks (left fist, right fist, both fists, and both feet) of each subject as T1, T2, T3, and T4, respectively. Since each MI task is slightly more than 4 s, we used a time window of 4s to unify the size of 4 MI tasks, and then performed 8–30 Hz band-pass filter processing for each MI task.

The positive problem of EEG is the use of EEG sensors to collect electrical signals generated by a large number of neurons in the brain (Wheless and Castillo, 2004). However, the signals transmitted from the brain to the scalp are already very weak and cannot accurately represent the activities inside the brain. Therefore, inverting EEG into the brain will improve the quality of EEG and also help improve its decoding intention. The process of using EEG to acquire signals inside the brain is an inverse problem (Becker, 2015). Solving the positive problem of EEG is the basis for solving its inverse problem.

The positive problem of EEG is also called the forward model of EEG, which can be described as follows (Baillet and Garnero, 1997; Engemann and Gramfort, 2015):


(1) $$\begin{array}{r} y=b+\varepsilon =Lx+\varepsilon \end{array}$$

Where *n* represents the number of sensors of EEG, *p* represents the number of dipoles in source space, *L*∈*R*^*n* × *p*^ is gain matrix or leadfield, and ε is noise.

According to Maxwell equations (Noraini et al., 2013), the electromagnetic field *R*^*n*^ in Formula (1) is the linear combination of the fields generated by all sources *x*∈*R*^*p*^:*b* = *Lx*. Solving the *L* matrix in Equation (1), finding sources that can best explain the value of EEG, and tracing the neurons in the brain is called the inverse problem of EEG (Janati et al., 2020).

The solution of the EEG forward problem consists of two parts: the head model and the algorithm. The head model is obtained by magnetic resonance imaging (MRI) of each subject. Since there is no permission to access the MRI of each subject, we used the high-precision colin27 template to build the head model (Collins et al., 1998).

In this part, we first completed the calibration of MRI and EEG and then calculated the noise covariance matrix according to baseline data for each subject to solve the problem of noise differences between different subjects. Then we used the BEM (Mosher et al., 1999; Gramfort, 2010) to solve the EEG positive problem and built a three-layer (cortex, skull, and scalp) head volume conduction model, also *L* was solved in the Equation (1).

Since the number of sources in the brain is far greater than the number of EEG sensors on the scalp, the result of the EEG inverse problem is not unique. It requires us to limit the source to a certain range. There are many cells on the cortex layer, they are close to the scalp, and the direction is basically perpendicular to the scalp, which is the main source of EEG (Okada, 1993). According to this, we used the DCD model to limit the source to the cortex layer of the brain. DCD model divides the entire cortex into discrete fixed sub-regions, each sub-region is placed with a current dipole perpendicular to the cortex, and this dipole is the source.

We used WMNE to solve the EEG inverse problem, as shown below (Phillips et al., 2002; Wu et al., 2003; Hassan et al., 2014):


(2) $$\begin{array}{r} I=L^{T}{\left(LL^{T}+\lambda \omega \right)}^{-1}R \end{array}$$

**Figure 2:**
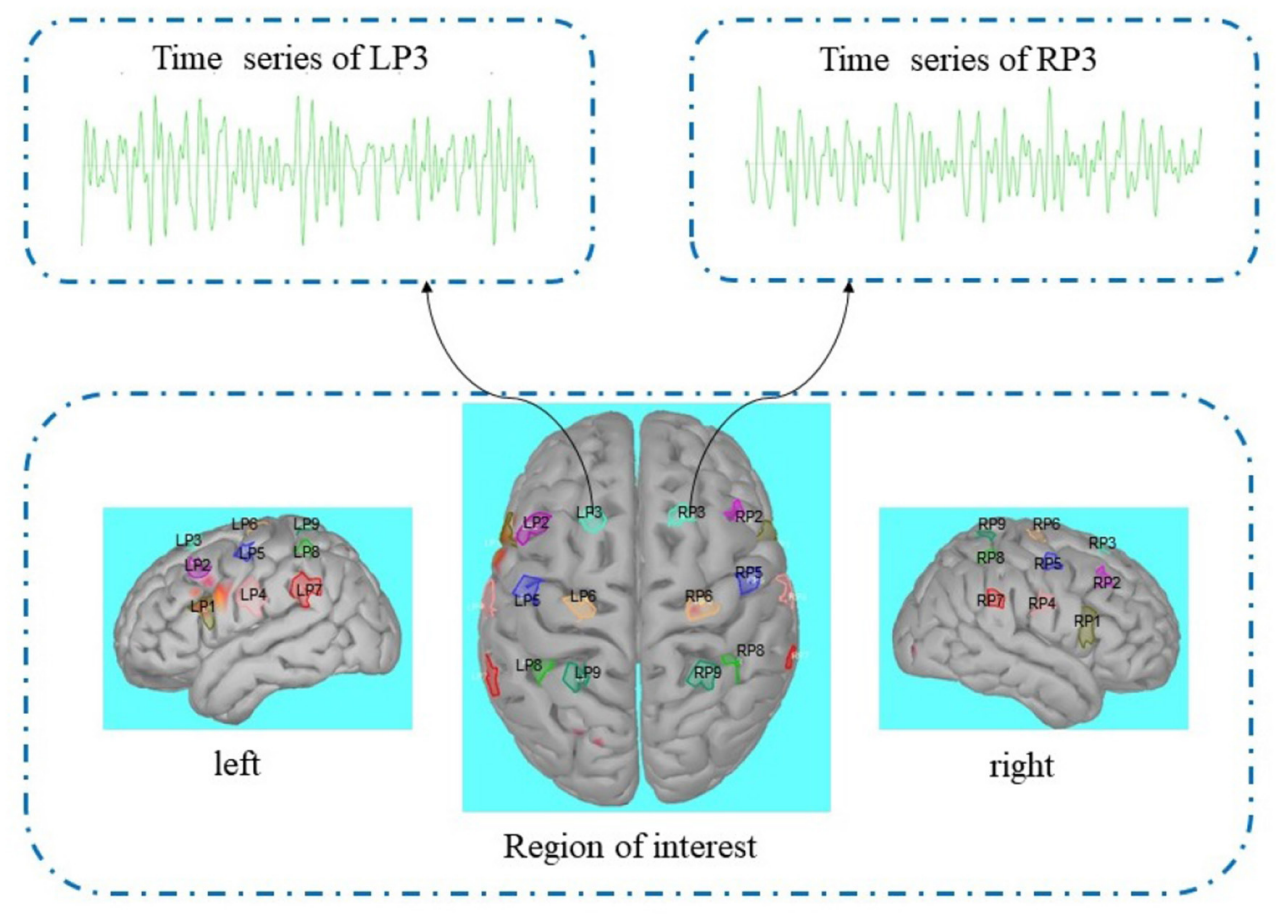
Cortex preprocessing.

Then we got a source model, the preprocessing of the cortex layer on the source model is shown in Figure 2. Similar to the research method of EEG source imaging, the region of interest can be selected by identifying particular gyral landmarks on the subject special cortex model (Edelman B. J. et al., 2015). According to Lun et al. (2020) we selected 18 scouts on the motor cortex as the region of interest. Nine sources on the left hemispheric are termed FC5, FC3, FC1, C5, C3, C1, CP5, CP3, CP1, and nine sources on the right hemispheric are termed FC6, FC4, FC2, C6, C4, C2, CP6, CP4, CP2. Each of the scouts was extended to 20 vertices, each vertex with one source (dipole) in constrained dipoles orientations. While the positions of the 18 sources are the projection on the cortex of nine pairs of electrodes (FC5, FC3, FC1, C5, C3, C1, CP5, CP3, CP1; FC6, FC4, FC2, C6, C4, C2, CP6, CP4, CP2), we marked these 18 sources as nine pairs of new electrodes on the left and right hemispheric. The time series of nine pairs of new electrodes on the left and right hemispheric were extracted by brainstorming in MATLAB (Tadel et al., 2011).

### 2.4. CNN Theory and CNN Structure

#### 2.4.1. CNN Theory

Convolutional neural network is generally composed of a convolution layer, pooling layer, and fully connected layer to complete feature extraction and classification (Schirrmeister et al., 2017; Kaldera et al., 2019). When the convolution operation is performed layer by layer, CNN can not only automatically extract rich features but also convey depth information. The initial layer of convolution is used to extract local features, and the end layer is used to extract global features. Among them, the convolutional layer contains multiple filters to extract features that are useful for classification (Liu and Liu, 2017). It uses the output of the previous layer as the input of the next layer to extract features, as follows (Ji et al., 2013):


(3) $$\begin{array}{r} x_{j}^{l}=f\left(\underset{i\in M_{j}}{\sum }x_{i}^{l-1}*k_{ij}^{l}+b_{j}^{l}\right) \end{array}$$

Where $x_{j}^{l}$ is the output of the jth channel of the l layer in the convolutional layer, *f*(·) is the activation function, *M*_*j*_ is the set of selection inputs, and $x_{i}^{l-1}$ is the output of the ith channel of *l*−1 layer in the convolutional layer, * represents convolution operation, $k_{ij}^{l}$ is convolution kernel matrix, and $b_{j}^{l}$ is offset value.

The pooling layer is generally used after the convolutional layer to reduce the number of parameters. It mainly includes average pool and max pool, which can be described as follows:


(4) $$\begin{array}{r} x_{j}^{l}=f\left(\beta_{j}^{l}down\left(x_{j}^{l-1}\right)+b_{j}^{l}\right) \end{array}$$

Where *f*(·) is the activation function, *down*(·) is the down-sampling function, $\beta_{j}^{l}$ is the weight coefficient of the jth channel of the lth layer in the pooling layer, and $b_{j}^{l}$ is bias (Zang et al., 2020).

Rectified linear unit (ReLU) is a commonly used activation function in convolution and pooling layers, which plays an important role in simulating biological neurons (Nair and Hinton, 2010; Karthik et al., 2020).

After multiple convolutional layers and pooling layers, the data will enter a fully connected layer. First, the data is processed by weighted summation, then processed by the activation function, and finally, the output of the fully connected network is obtained, as follows:


(5) $$\begin{array}{r} x^{l}=f\left(\omega^{l}x^{l-1}+b^{l}\right) \end{array}$$

Among them, ω^*l*^*x*^*l*−1^+*b*^*l*^ is the net activation of layer l in a fully connected layer. ω^*l*^ is the weight coefficient, and *b*^*l*^ is bias.

Since the training is for subjects, the output of each category label will be converted into conditional probability by the softmax function as follows (Amin et al., 2019):


(6) $$\begin{array}{r} p\left(l_{k}|f\left(X^{j};\theta \right)\right)=\frac{expf_{k}\left(X^{j};\theta \right)}{\sum_{k=1}^{K}exp\left(f_{k}\left(X^{j};\theta \right)\right)} \end{array}$$

Where *l*_*k*_ is the given label, *X*^*j*^ is input, θ is parameter including weight and bias, and *K* is the category.

#### 2.4.2. CNN Structure

The network structure and parameters of CNN are determined by the experimental method, as shown in Table 1. We proposed a novel dual-CNN model for MI classification, which can process the time series of nine hemispheric electrodes on the cortex layer, and the structure is shown in Figure 1. The specific process description is as follows:

1. We used 4s MI data as the input of the neural network. At a sampling frequency of 160Hz, its data dimension is 640, and the dimension remains unchanged after cortex layer processing. First, connect the time series of the left and right hemispheric symmetrical electrodes horizontally, and then connect the data of the nine pairs of electrodes vertically, so that the data format that enters the neural network is 1, 280 × 9. The first layer of the network separates the nine electrodes of the left and right brains through the reshape operation to form two data with a size of 640 × 9, representing the time series of the nine electrodes of the left and right brains, respectively.
2. We used two CNN structures with exactly the same parameters to form a dual-CNN model. Each CNN structure contains 4-layer CNN for learning features, 4-layer max pooling for dimensionality reduction, and 1 FC layer that converts multi-dimensional data into one dimension.
3. The one-dimensional data output by the left and right CNN model are subtracted, then the signal differences of the symmetrical electrode are entered into the FC layer, and the softmax function is used to predict the attribution of the test data.

In addition, based on 4-layer CNN and 4-layer max pooling, we try to add more CNN layers and max pooling layers. It is found that 4-layer dual-CNN performs best in the experiment, and the accuracy is not significantly improved after the number of convolutional layers exceeds 4-layer.We used spatial dropout and batch normalization (BN) techniques to prevent overfitting. In Section 3.5, our proposed model is compared with other models, and a better classification evaluation effect is obtained.

**Table 1:** Proposed CNN architecture.

	**Layer**	**Input size**	**Map**	**Convolution**	**Pooling**	**Output size**
				**kernel size**	**size**	
L1	Input	1,280×9	1	-	-	640×9,640×9
L2	Conv_L1,Conv_R1	640×9	25	11×9×25	-	630×1×25
L3	Pool_L1, Pool_R1	630×1×25	25	-	3×1	210×1×25
L4	Conv_L2,Conv_R2	210×1×25	50	11×1×50	-	200××50
L5	Pool_L2, Pool_R2	200×1×50	50	-	3×1	66×1×50
L6	Conv_L3,Conv_R3	66×1×50	100	11×1×100	-	56×1×100
L7	Pool_L3, Pool_R3	56×1×100	100	-	3×1	18×1×100
L8	Conv_L4,Conv_R4	18×1×100	200	11×1×200	-	8×1×200
L9	Pool_L4, Pool_R4	8×1×200	200	-	2×1	4×1×200
L10	Flatten_L,Flatten_R	4×1×200	1	-	-	800
L11	Flatten_L-Flatten_R	800	1	-	-	800
L12	FC	800	1	-	-	128
L13	Softmax	128	1	-	-	4

## 3. Results

### 3.1. Classification Accuracy of Individual Subject

In order to obtain effective results, events T1-T4 in each subject are randomly intermingled and separated into 90% as the training set, and the remaining 10% as the test set. We conducted trial-based accuracy experiments for each subject (S1-S10) on the Physionet database. Table 2 lists the accuracy of each subject and its four MI tasks (T1, T2, T3, T4). In Table 2, the highest accuracy is 98.88% (S4), and its four MI accuracies are 99.62, 99.68, 98.47, and 97.73%, respectively. The lowest accuracy is 96.23% (S7), and its four MI accuracies are 99.14, 93.18, 96.45, and 96.15%, respectively. The average accuracies of the four MI tasks for ten subjects are 99.57% (T1), 97.30% (T2), 95.70% (T3), and 96.92% (T4), respectively. T1 has the highest accuracy, it is indicative that the classification effect of the left fist is the best. The accuracy of T3 is the lowest, it indicates that the classification effect of both fists is the worst. The highest accuracy on T1 is 99.92% (S8), while the lowest is 98.93% (S6). The highest accuracy on T2 is 99.68% (S4) and the lowest is 93.18% (S7). The highest accuracy on T3 is 99.56% (S10), while the lowest is 90.91% (S8). The highest accuracy on T4 is 99.34% (S5), while the lowest is 92.86% (S3). According to the above results, it can be found that our proposed method achieves higher accuracy on S1-S10 and its four MI tasks.

**Table 2:** The classification accuracy of individual subject.

**Subject**	**Accuracy (%)**	**T1 accuracy (%)**	**T2 accuracy (%)**	**T3 accuracy (%)**	**T4 accuracy (%)**
S1	97.77	99.67	97.14	96.08	98.18
S2	97.30	99.59	96.00	96.45	97.17
S3	96.35	99.73	96.90	95.89	92.86
S4	98.88	99.62	99.68	98.47	97.73
S5	97.14	99.56	98.15	91.49	99.34
S6	97.61	98.93	97.56	95.91	98.04
S7	96.23	99.14	93.18	96.45	96.15
S8	96.33	99.92	99.37	90.91	95.12
S9	97.34	99.81	97.44	95.83	96.27
S10	98.81	99.74	97.56	99.56	98.36

**Figure 3:**
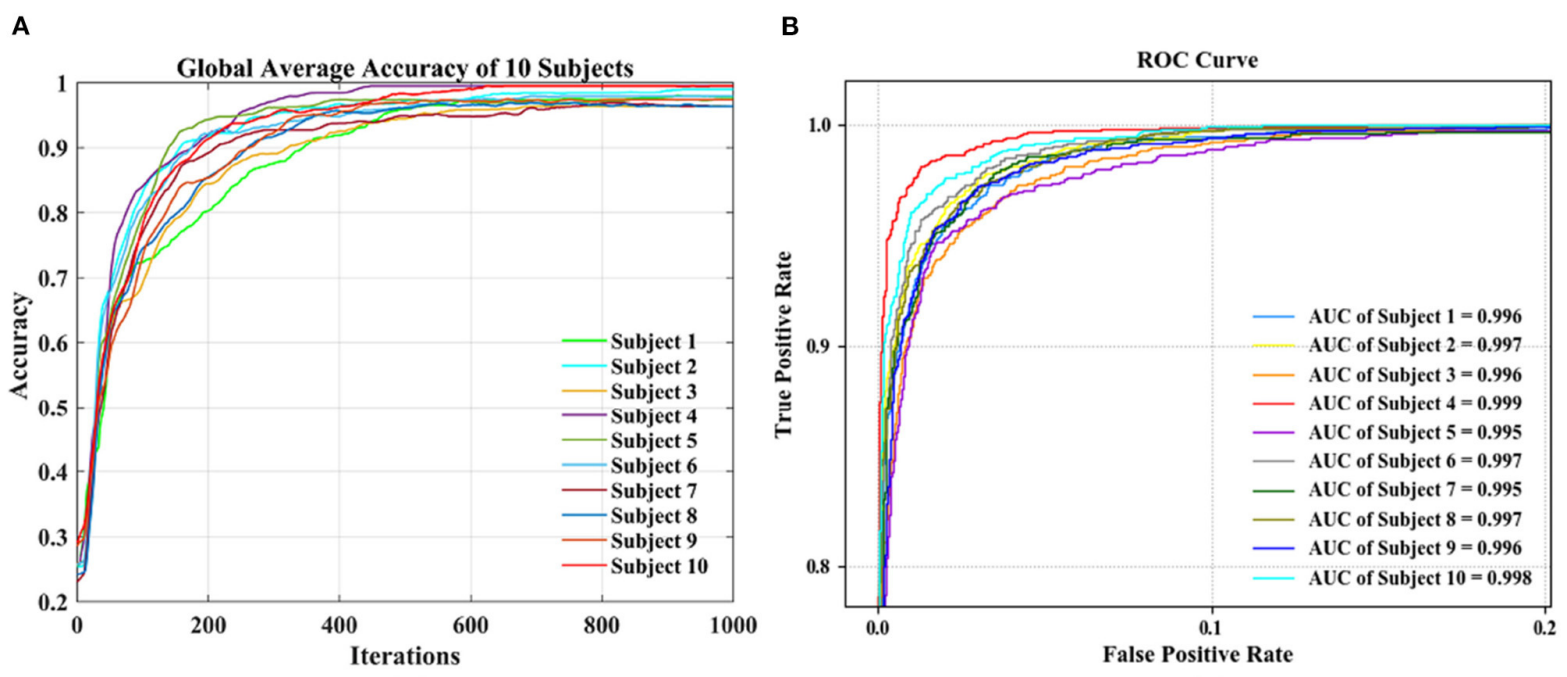
Performance comparison of 10 subjects. **(A)** Accuracy comparison. **(B)** Receiver operating characteristic (ROC) curve comparison.

During the CNN training iteration process, the accuracy curves of the ten subjects are shown in Figure 3A. The accuracy of an individual subject increases rapidly during the first 200 iterations and slows down during the 200–600 iterations. After 600 iterations, the accuracy can reach a stable state. The accuracy curve of the whole iterative process is relatively smooth with less burr. It shows that as the number of iterations increases, our proposed dual-CNN can achieve high classification accuracy and high stability.

The receiver operating characteristic (ROC) curves of 10 subjects are shown in Figure 3B, which is used to evaluate the classification model. The area under the ROC curve is represented by AUC, and the value range is between 0.5 and 1. The closer the AUC is to 1.0, the better the classification effect is. Among the 10 subjects, the best classification model is S4, with an AUC value of 0.999, and the worst classification model has an AUC value of 0.995 (S7). It can be seen the proposed method has achieved better generalization performance and higher classification effect in different subjects.

### 3.2. Classification Accuracy of Group-Level Subjects

**Figure 4:**
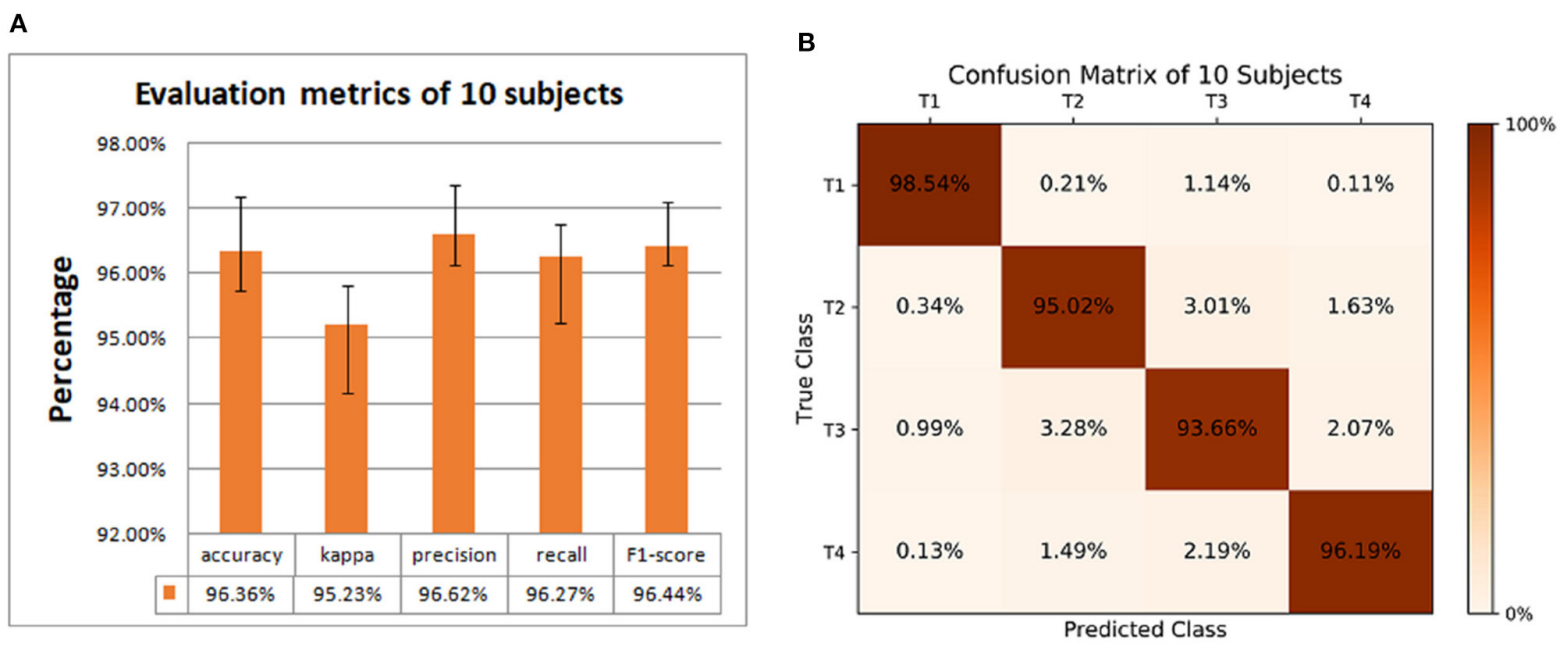
Classification performance of 10 subjects. **(A)** Evaluation metrics. **(B)** Confusion matrix for the accuracy of 4 motor imagery (MI) tasks.

We also conducted a group-level experiment of 10 subjects to obtain the classification performance. In this part, first, we divided the data of each subject into two groups: the training set and the test set. T1-T4 of each subject are randomly divided into 10 equal parts, 9 parts are mixed uniformly to become the training set, and the remaining part is randomly shuffled into the test set. The training set of each subject is mixed to form the final training set, and the test set of each subject is mixed to form the final test set. Then we used five index evaluations to measure the effectiveness of classification, as shown in Figure 4A, accuracy, kappa, precision, recall, and F1-score are 96.36, 95.23, 96.62, 96.27, and 96.44%, respectively.

The confusion matrix in Figure 4B shows the accuracy of the 4 MI tasks at the group-level. The values on the diagonal of the confusion matrix are the correct classification, and the other values are the wrong classification. The accuracies of 4 MI tasks are 98.54 (T1), 95.02 (T2), 93.66 (T3), and 96.19% (T4), respectively. It can be seen the proposed method can also achieve good performance in group-level classification.

### 3.3. Comparison of Classification Models

In order to solve the problem of overfitting, spatial dropout and BN were used in our proposed model. Dropout refers to the random “temporary dropping” of a part of neuron nodes with a certain probability in training. Different neurons are then combined with each other for optimization during each training process. This process weakens the joint adaptability of all neurons and reduces the risk of overfitting. BN enhances the generalization ability of the model by imposing additional constraints on the distribution of data.

In this article, we compared the performance of our proposed models with three different models based on the data set of 10 subjects at group-level. Table 3 compared the performance of the four models with five evaluation indicators. The accuracy, kappa, precision, recall, and F1-Score of our proposed model are 96.36, 95.23, 96.62, 96.27, and 96.44%, respectively, which are higher than other models, so its performance is better than other models.

**Table 3:** Performance comparison of different convolutional neural network (CNN) models.

**Model**	**Accuracy (%)**	**kappa (%)**	**Precision (%)**	**Recall (%)**	**F1-score (%)**
Proposed model	96.36	95.23	96.62	96.27	96.44
Model without dropout	94.06	90.74	94.32	93.82	94.07
Model without BN	90.77	89.02	90.51	91.20	90.85
Model without	86.39	82.24	86.77	86.13	86.45
dropout & BN					

**Figure 5:**
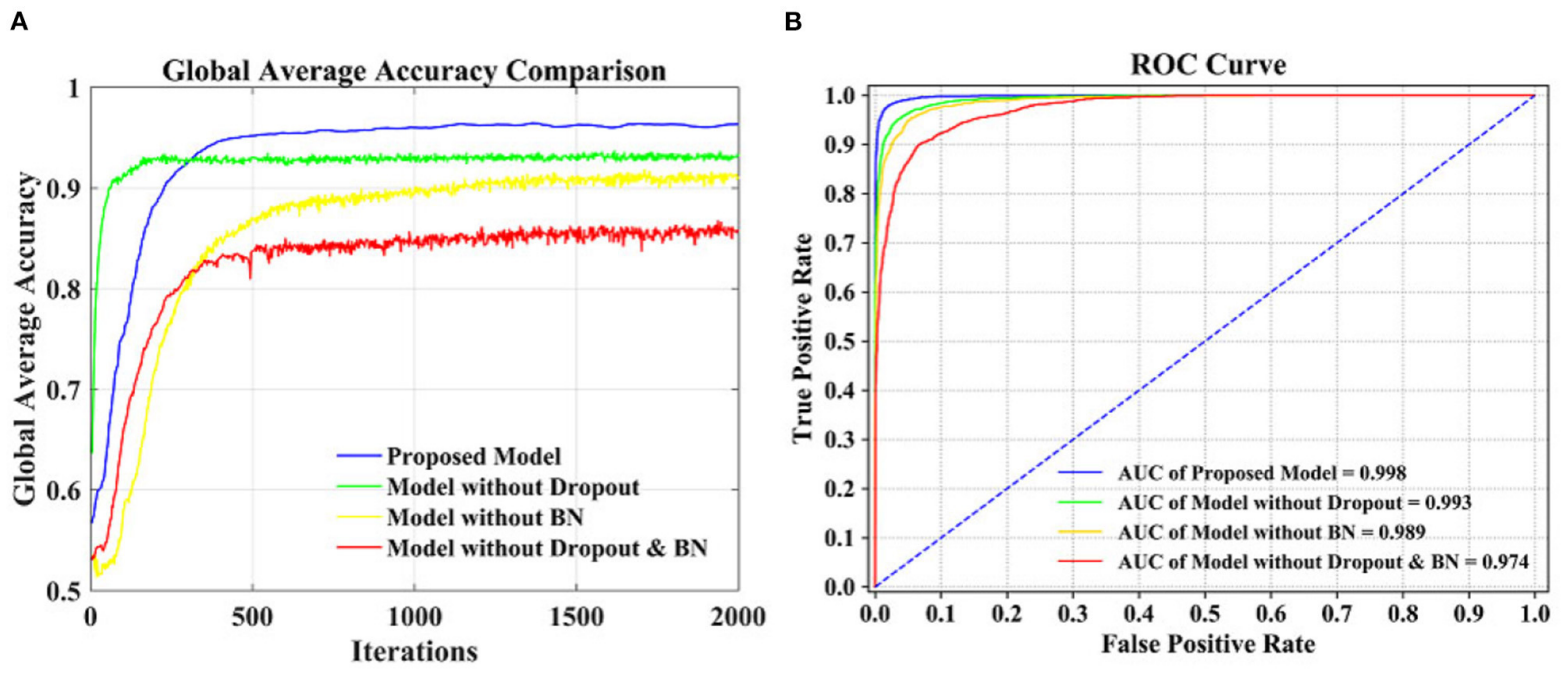
Performance comparison of different models. **(A)** Accuracy comparison. **(B)** ROC curve comparison.

Figure 5A is a comparison of the global average accuracy of the four models. It can be seen that all models can reach a stable state after iteration. Currently, the proposed model has the highest accuracy of 96.36%, followed by 94.06% for the model without dropout, 90.77% for the model without BN, and 86.39% for the model without dropout and BN. Figure 5B is the ROC curve and AUC curve of the four models. The model proposed in this paper has the largest AUC value, 0.996, which is the closest to 1, and the classification effect is the best. The accuracy curve and ROC curve of the CNN model we proposed to solve the overfitting problem are the smoothest and with the smallest burr. In addition, the values of various evaluation metrics are the highest, and the AUC value is also the highest when reaching the stable state after iterations. When the iteration reaches a stable state, the five evaluation indicators of accuracy, kappa, precision, recall, and F1-score are all the highest, and the AUC value is the largest. The performance of our proposed model is the most stable, and it does improve the classification effect.

### 3.4. Comparison of Loss on Test Data

**Figure 6:**
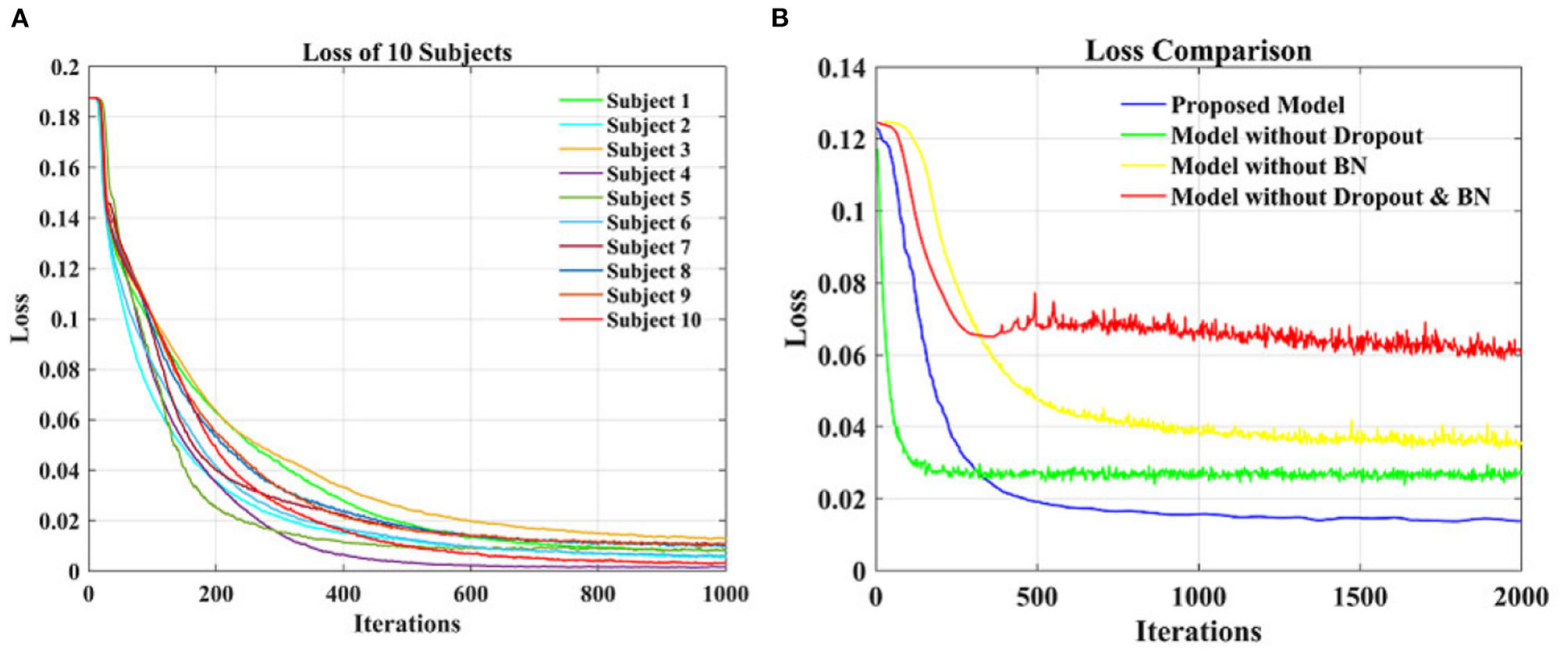
The loss function curve on test data. **(A)** Loss function comparisons of 10 individual subjects. **(B)** Loss function comparisons of different classification models.

Figure 6A shows the loss function curve of ten individual subjects on the test set, whose loss values decrease with the increase of iteration times. When the number of iterations is about 600, the loss values remain basically stable. Thus, the optimal testing effect can be obtained, and it can be seen that our model is convergent during testing. Figure 6B shows a comparison of the loss function curve of different classification models on the test set of group-level subjects. The loss values of the four curves decrease with the increase of iteration times and can reach equilibrium after 500 iterations. The blue curve is the test loss function curve of the proposed model. Compared with the other three models, it has the smoothest curve, the smallest burr, and the smallest loss when it reaches the stable state. In general, the proposed model has a good convergence effect on the test set of the individual subject and group-level subjects.

### 3.5. Comparison With Other Works

Electroencephalogram signal has low amplitude and contains a lot of noise, and there are differences between different subjects. This article compared and analyzed our study with Handiru and Prasad (2016), Azimirad et al. (2017), Dose et al. (2018), Athif and Ren (2019), and Hou et al. (2019) in Table 4 under the same database and the same MI task. The results show that our method achieved the best results on both group-level subjects and individual subjects, which indicates that the difference between the left and right hemispheric on the cortex contains more information related to MI tasks and that our CNN structure is very helpful in improving the generalization performance of the model.

**Table 4:** Performance comparison with other studies.

**Work**	**Training**	**Accuracy (%)**	**Methods**
Azimirad et al. (2017)	Global	81.00	SVM
Dose et al. (2018)	Global	80.38	CNN
	Subject	86.49	
Athif and Ren (2019)	Global	64.00	CSP
Hou et al. (2019)	Global	94.54	ESI + CNN
	Subject	94.50	
Handiru and Prasad (2016)	Global	61.01	SVM
This work	Global	96.38	CNN
	Subject	98.88	

In particular, the subjects used in the Hou et al. (2019) partially overlap with the subjects used in our article, which are S5-S10. When the single subject is tested, the highest accuracies in Y.Hou et al. are 94.6%(S5), 94.1%(S6), 95.0%(S7), 93.2%(S8), 95.5%(S9), and 93.1%(S10). Then the accuracies of the method proposed in this article are 97.14%(S5), 97.61%(S6), 96.23%(S7), 96.33%(S8), 97.34%(S9), and 98.81%(S10), which are higher than Hou et al. In terms of real-time performance comparison, none of the articles achieved real-time control.

## 4. Discussion

### 4.1. Data Analysis

From Table 2 it can be found that the average accuracy of a single subject in this article is up to 98.88%, which is an improvement of 12 and 4% respectively compared with Dose et al. (2018) and Hou et al. (2019). This proves the effectiveness of this method. Specifically, Dose et al. only processes raw EEG signal, while this article processes the region of interest on the cortex layer, which shows that the preprocessing operation in this article is effective. Hou et al. used single layer CNN to classify data, which illustrates the feasibility and reliability of our proposed dual-CNN. Figure 4 shows that the highest global accuracy rate of this article is 96.38%, which is also higher than Dose et al. and the Hou et al. In addition, our group-level accuracy is also higher than other articles in Table 4, which proves that the dual-CNN proposed in this paper has a significant effect on MI-BCI classification.

### 4.2. Limitations of the Proposed Method

The time spent on data processing and classification using neural networks is related to the amount of data, the complexity of the network structure, and the performance of computer equipment. When using CNN to process MI data, each learning iteration during the CNN training will take some time. At present, this paper cannot detect and classify MI tasks in real-time. Therefore, Figure 3 still uses learning iterations during CNN training, instead of using time as the abscissa axis. But it is meaningful that the number of iterations is also another manifestation of time, which means that as time increases, our classification accuracy will continue to increase and eventually reach a stable state. With the improvement of computer performance, it will take less and less time for us to reach a stable state. Therefore, in the classification of non-real-time MI-BCI, it is also a good way to display the performance of the method with the number of iterations. There is still scope for simplifying the network structure, which will be investigated in the future. Also, it would be interesting in the future to employ the current method for real-time online BCI experiments.

## 5. Conclusion

The key objective of the study presented in this article is to investigate the method of high classification accuracy on MI-EEG signals. This article proposed a new MI classification method that combines the difference between the left and right hemispheric electrodes on the cortex and dual-CNN. Using the Physionet database as the data source, restored the raw EEG signal from the low-density EEG scalp measurement, mapped nine pairs of electrodes from the scalp layer to the cortex layer as the region of interest, and extracted the time series of nine pairs of electrodes signals as the input of the proposed dual-CNN classification model. The results demonstrated that these MI tasks can be classified with high accuracy by the difference between the signals left and right hemispheric electrodes, and CNN plays an important role in improving generalization performance. The BCI system of MI based on left and right hemispheric electrodes and CNN can be applied in the daily life of all subjects. The results suggested that the classification accuracy of the proposed method is substantially higher than all other methods used in this study.

## Data Availability Statement

Publicly available datasets were analyzed in this study. This data can be found at: https://physionet.org/content/eegmmidb/1.0.0/.

## Author Contributions

XL: method design of the article. JL: programming and software design. YZ: data collection and pre-processing. ZH: experimental improvement and paper revise. YH: result analysis, method improvement, and manuscript calibration. All authors contributed to the article and approved the submitted version.

## Conflict of Interest

The authors declare that the research was conducted in the absence of any commercial or financial relationships that could be construed as a potential conflict of interest.

## Publisher's Note

All claims expressed in this article are solely those of the authors and do not necessarily represent those of their affiliated organizations, or those of the publisher, the editors and the reviewers. Any product that may be evaluated in this article, or claim that may be made by its manufacturer, is not guaranteed or endorsed by the publisher.
